# Investigation into the Use of Microfluidics in the Manufacture of Metallic Gold-Coated Iron Oxide Hybrid Nanoparticles

**DOI:** 10.3390/nano11112976

**Published:** 2021-11-05

**Authors:** Adeolu Oluwasanmi, Ernest Man, Anthony Curtis, Humphrey H. P. Yiu, Yvonne Perrie, Clare Hoskins

**Affiliations:** 1 Department of Pure and Applied Chemistry, University of Strathclyde, Glasgow G1 1RD, UK; adeolu.oluwasanmi@strath.ac.uk (A.O.); ernest.man@strath.ac.uk (E.M.); 2 School of Pharmacy and Bioengineering, Keele University, Keele ST5 5BG, UK; a.d.m.curtis@keele.ac.uk; 3 Chemical Engineering, School of Engineering and Physical Sciences, Heriot-Watt University, Edinburgh EH14 4AS, UK; h.h.yiu@hw.ac.uk; 4 Strathclyde Institute of Pharmacy and Biomedical Sciences, University of Strathclyde, Glasgow G4 0RE, UK; yvonne.perrie@strath.ac.uk

**Keywords:** hybrid nanoparticle, manufacture, scale up, microfluidics, nanotechnology

## Abstract

Hybrid iron oxide-gold nanoparticles are of increasing interest for applications in nanomedicine, photonics, energy storage, etc. However, they are often difficult to synthesise without experience or ‘know-how’. Additionally, standard protocols do not allow for scale up, and this is significantly hindering their future potential. In this study, we seek to determine whether microfluidics could be used as a new manufacturing process to reliably produce hybrid nanoparticles with the line of sight to their continuous manufacture and scaleup. Using a Precision Nano NanoAssemblr Benchtop^®^ system, we were able to perform the intermediate coating steps required in order to construct hybrid nanoparticles around 60 nm in size with similar chemical and physical properties to those synthesised in the laboratory using standard processes, with Fe/Au ratios of 1:0.6 (standard) and 1:0.7 (microfluidics), indicating that the process was suitable for their manufacture with optimisation required in order to configure a continuous manufacturing plant.

## 1. Introduction

Whilst many nanoparticles have shown excellent potential in laboratory testing for sectors such as energy [1], agriculture [2] or healthcare [3], the ability to reliably and repeatably scale up their manufacture has hindered their ability to be marketed or proceed further down their developmental pipeline, particularly for clinical application [4,5,6]. In the past 5 years, an abundance of work has been reported on the scale up and continuous manufacture of liposomes using microfluidics [7,8,9,10,11]. Microfluidics allows for highly defined shear mixing of two or more compounds in highly tailorable chambers, which can be designed bespoke to the platform being developed [12]. Here, mixing rate, duration and type are controllable, as well as the ability to consecutively feed starting materials into the inlet ports in order to form a continuous system, thus allowing for larger-scale manufacture [12]. Liposome studies have shown that using microfluidics, large-scale batches can be formed, which do not compromise on particle quality [13]; this not only includes liposome macromolecular assembly, but also cargo such as protein drugs or other compounds of interest.

**Figure 1:**
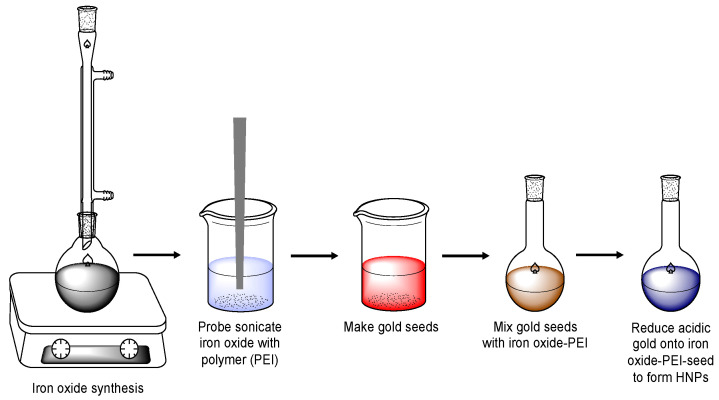
Current benchtop synthesis route to form core-shell gold-coated iron oxide hybrid nanoparticles.

Hybrid iron oxide-gold nanoparticles (HNPs) have shown to be promising platform technologies, particularly within healthcare technology, where they have been reported as diagnostic tools [14], drug delivery systems [15] and theranostics [16]. Here, the combination of a magnetic core from the iron oxide alongside the plasmonic properties and biocompatibility of the gold shell offers an exciting multifunctional system, particularly in areas such as cancer nanomedicine [17]. HNPs can be fabricated on small scales of up to 20 mL reliably [18]. The synthetic route for these particles firstly requires a simple coprecipitation to form the iron oxide core. These are subsequently coated with a long-chain cationic polymer before electrostatically attaching 2 nm gold seeds onto their surface. Finally, the complete gold coating is achieved using the iterative reduction of chloroauric acid (Figure 1) [18]. Parameters such as polymer interaction, core size and coating thickness and their effect on physicochemical properties have previously been reported [18]. The multistep benchtop synthesis of HNPs can often be complicated with the lack of in-depth knowledge and experience, leading to unreliable batch-to-batch variation caused by human error or contamination causing issues. Nevertheless, once the hybrids have been formed and are known to be of good quality, their strength in application is undisputable. Therefore, in order to allow these HNPs to reach their full potential and progress towards clinical trials for healthcare, or market for energy storage, large-scale manufacture must first be possible. Attempts to scale up the benchtop chemistry have thus far not harnessed useful developments; therefore, the use of microfluidics will be evaluated.

The synthesis of inorganic nanohybrids using a microfluidic system was recently discussed for the formation of Au@CoFeB-Rg3 nanomedicines for their anti-tumour effects [19]. The synthesis of iron oxide nanoparticles on a millifluidic platform was also recently reported, with an internal channel diameter of 1 mm [20]. At the microscale, however, precipitation-based reactions may be prone to deposition and clogging within flow-based systems. In this study, we seek to determine whether the chemical synthesis of core-shell gold-coated iron oxide particles is feasible using microfluidics, a possibility that has been recently discussed in the literature [21]. Additionally, very recently, Ahrberg and colleagues reported the synthesis of iron oxide coated with gold hybrid nanoparticles using a droplet reactor [22]. This important study paves the way for further work in the field, where modification of the iron coating is required such as our particles, where a poly(ethylenimine) intermediate layer is added in order to help maintain the integrity of the physical properties of both the iron oxide and gold shell for later applications.

The process of HNP synthesis usually relies on high temperatures and lengthy reaction durations for crystal formation. However, this investigation will allow us to elucidate whether the superior mixing of microfluidics could be used to more efficiently produce these. Secondly, we will investigate whether microfluidics can be used in the stepwise coating required for HNP assembly with a view to recommend whether or not this technology has potential for the future development of large-scale batches of these particles. All studies will be compared to the current benchtop synthetic procedure for HNP formation.

## 2. Materials and Methods

### 2.1. Hybrid Nanoparticle Synthesis

HNPs were synthesised as previously reported [15,16,18]. Briefly, Fe_3_O_4_ nanoparticles were first synthesised using a coprecipitation reaction between iron sulphate heptahydrate (Sigma Aldrich, UK) and sodium hydroxide/potassium nitrate (Sigma Aldrich, UK) in acidic conditions under nitrogen and 90 °C. The mixture was left to stir for 24 h under reflux before the mixture was plunged into icy water to stop further crystallisation. The particles were washed with deionised water before sonication with poly(ethylenimine) (Sigma Aldrich, Gillingham, UK) for 2 h. Gold seeds were formed by the reduction of chloroauric acid (Sigma Aldrich, Gillingham, UK) with sodium carbonate (Sigma Aldrich, Gillingham, UK) and sodium borohydride (Sigma Aldrich, Gillingham, UK). The gold seeds were stirred with the polymer-coated iron oxide particles for 2 h at 25 °C before washing. The gold coating was obtained by further iterative reduction of chloroauric acid onto the surface using hydroxylamine (Sigma Aldrich, Gillingham, UK). Once the HNPs had formed, the particles were washed and stored at 25 °C until further use.

### 2.2. Microfluidic Hybrid Nanoparticle Synthesis

The NanoAssemblr Benchtop system (Precision Nanosystems, Vancouver, Canada) was operated with Precision Nano’s proprietary software (Precision Nanosystems, Vancouver, Canada) that removed batch-to-batch and user variability. This system is marketed by Precision Nano to reproducibly produce lipid nanoparticles, liposomes, polymeric nanoparticles, emulsions and metallic nanoparticles. This system has an operational working volume of 1–15 mL, with two syringe input ports directly situated on the commercially available NanoAssemblr^®^ Benchtop cartridges (Figure S1), each independently fed by separate syringe infusion pumps. The first and last 2.5% of the output were collected separately for waste with each run. The NanoAssemblr^®^ Benchtop cartridges contained etched channels designed to provide reproducible laminar mixing of both input solutions.

Throughout the synthesis route, magnetic separation is utilised to separate magnetic nanoparticles from contaminants and excess unused reagents. This is achieved by placing a powerful magnet against the NanoAssemblr Benchtop output collection vial walls for 5 min prior to decanting.

The process of manually loading both syringes, fitting them to the NanoAssemblr system, initiating the computer-controlled mixing process and, after completion, removing both syringes and the output vial required approximately 92 s. Compensating for the reagent solutions of the microfluidics system being 100 times more dilute than with the benchtop procedure, the time required (initially from uncoated IONPs) to produce the same quantity of HNPs is approximately 153 min when operated continuously. This could be further reduced by replacing the human element of manual syringe loading and output vial removal with syringe pumps for a reagent solution stock and a large reservoir to collect the output. This would also mitigate any errors arising from human input that may arise. In contrast, the benchtop procedure of producing HNPs from uncoated IONPs requires 2 h of sonication, 2 h of gold seed loading and 10 min/reagent addition cycle +30 min of further stirring, leading to approximately 7 h total. Therefore, the continuous use of the NanoAssemblr system would yield nanoparticles at 2.75 times the rate of the standard benchtop method.

#### 2.2.1. Synthesis of Fe_3_O_4_

After heating all solutions to 90 °C under nitrogen flow, a 120 mM/100 mM aqueous solution of sodium hydroxide/potassium nitrate (9 mL) was mixed with a 1.3 M solution of iron sulphate heptahydrate in 0.01 M sulfuric acid using the NanoAssemblr Benchtop^®^ system (Precision Nanosystems, Vancouver, Canada) in a 9:1 ratio at a rate of 10 mL/min.

#### 2.2.2. PEI Coating of Fe_3_O_4_

Fe_3_O_4_ nanoparticles (18.87 mgmL^−1^ Fe) were diluted by a factor of 100 in deionised water. The diluted suspension (0.189 mgmL^−1^ Fe, 10 mL) was mixed with a solution of PEI 750 k, 5 mgmL^−1^ (2 mL), within the microfluidic cartridge in a 5:1 ratio at a rate of 10 mL/min. The output solution was magnetically separated and washed 5 times with 10 mL deionised water before being suspended in 1 mL of deionised water.

#### 2.2.3. Gold Seeding of Fe_3_O_4_-PEI

Fe_3_O_4_-PEI (7.36 mgmL^−1^ Fe) was diluted by a factor of 100 in deionised water (10 mL) and was mixed with a 2 nm gold seed solution (prepared in the laboratory, 2 mL) within the microfluidic cartridge in a 5:1 ratio at a rate of 10 mL/min. The output solution was magnetically separated and washed 5 times with 10 mL deionised water before being suspended in 1 mL of deionised water. Gold seeds were prepared in the laboratory by dissolving chloroauric acid HAuCl_4_ (375 µL, 4%) and Na_2_CO_3_ (500 µL, 0.2 M) in 100 mL DI water, which was chilled to 5 °C and stirred for 10 min. NaBH_4_ (0.5 mgmL ^−1^, 5 mL) was freshly prepared in DI water at 5 °C and added in 1 mL/min portions, leading to the formation of a deep red solution of gold seeds that was allowed to stir for a further 10 min prior to immediate use.

#### 2.2.4. Gold Coating of Fe_3_O_4_-PEI-Au Seeds

Fe_3_O_4_-PEI-Au seeds (7.36 mgmL^−1^ Fe) were diluted by a factor of 100 in 0.012 mM sodium hydroxide. Chloroauric acid (1%, 50 µL) was added to the diluted suspension of Fe_3_O_4_-PEI-Au seeds (5 mL). This was subsequently mixed with a 0.2 M solution of hydroxylamine (75 µL) using the microfluidics cartridge in a 5.05:0.95 ratio at a rate of 10 mL/min. Further additions of 50 µL of 1% chloroauric acid and 0.2 M solution of hydroxylamine (25 µL) were added to the output solution iteratively with further mixing after each addition within the microfluidic cartridge; six iterative additions were added in total while maintaining a flow rate of 10 mL/min. The final output of HNPs was magnetically separated and washed 5 times with 10 mL deionised water before being suspended in 1 mL of deionised water.

### 2.3. Photon Correlation Spectroscopy

Benchtop and microfluidic-derived particles were diluted 1000- and 10-fold, respectively, in deionised water and sonicated in a sonic bath prior to analysis. Samples (1 mL) were placed into a disposable cuvette, and particle size was measured at 25 °C in a Malvern Nanosizer Zeta-DS (Malvern, UK). Samples were measured in triplicate with average values and standard deviations recorded.

### 2.4. Zeta Potential Measurement

Benchtop and microfluidic-derived particles were diluted 1000- and 10-fold, respectively, in deionised water and sonicated in a sonic bath prior to analysis. Samples (1 mL) were placed into a folded capillary cell, and particle surface charge was measured at 25 °C in a Malvern Nanosizer Zeta-DS (Malvern, UK). Samples were measured in triplicate with average values and standard deviations recorded.

### 2.5. Inductively Coupled Plasma—Optical Emission Spectroscopy

Samples were acid digested in a 1:1 hydrochloric acid/nitric acid solution with gentle heating. Once the samples had fully digested, they were further diluted with deionised water and analysed for Fe (238.204) and Au (242.794) content using an Agilent Technologies 700 series system (Agilent, Santa Clara, CA, USA). Samples were compared to a calibration pf 0–10 ppm, and the metal ratio Fe/Au was calculated.

### 2.6. UV–Vis Spectroscopy

Absorbance of samples in deionised water was measured using a Varian UV-Vis Cory 50 Bio spectrometer (Agilent, Santa Clara, CA, USA). Samples were analysed in quartz cuvettes, and absorbance scans were carried out between 400 and 800 nm.

### 2.7. Fourier Transform Infrared Spectroscopy

The samples were freeze dried into a powder form before being placed under the diamond tip of an ATR attachment of a Perkin Elmer (Perkin Elmer, Waltham, MA, USA). The samples were scanned 20 times following background correction.

### 2.8. Thermogravimetric Analysis

Analysis was carried out on a TA SDT Q600 (TA Instruments, New Castle, DE, USA) with sequences of 20 °C/min ramp to 90 °C, isothermal for 10 min and 20 °C/min ramp to 600 °C.

### 2.9. Transmission Electron Microscopy

Samples (20 µL) were pipetted onto formvar coated copper grids and left to air dry before analysis. Transmission electron micrographs were obtained using a JEOL JEM-1230 (JEOL, Tokyo, Japan). Samples were scanned over a large area and a representative image for the sample was obtained.

## 3. Results and Discussion

Currently, gold-coated iron oxide hybrids are synthesised in small batches as a benchtop multistep synthesis in the laboratory (Figure 1), which is monitored via measurement of zeta potential at each point of the synthesis to track progress. The successful completion of each step is judged by changes in surface charge, which was verified using Fourier transform infrared spectroscopy, UV-vis spectroscopy and thermogravimetric analysis at various stages. In this study, the possibility of using microfluidics as a technique for the manufacture of HNPs was investigated. One major challenge hindering the ability of these exciting HNP platforms as diagnostic agents, drug delivery vehicles and theranostic applications, for progressing further down the pathway towards clinical translation, is their ability to be manufactured at a large scale. Following on from the success in the use of microfluidics for liposomes, the ability to create core-shell HNPs was evaluated, with each synthetic step monitored with zeta potential measurements and compared with benchtop-derived NPs produced in parallel. As shown in Figure 1, the first step in the fabrication of these particles is to form the magnetic iron oxide (Fe_3_O_4_) nanoparticulate core. The benchtop procedure requires long durations, reaction under inert gas and high temperature in order to allow for crystal formation. To determine whether the superior mixing within the microfluidic chamber could expedite this process, the reactants were first heated to 90 °C before being fed through syringe drivers into the NanoAssemblr cartridge. Using the NanoAssemblr setup, it was not possible to heat the inlet tubing to the cartridge to maintain consistency of temperature. However, the transfer time was so rapid that we believe there would have been a negligible decrease before the reactants were mixed. Introduction of the iron sulphate with the base solution within the cartridge led to blockage of the chamber due to the rapid generation of particulates. Consistent with the iron oxide produced using the standard laboratory method, the particulates were black in colour, but unfortunately, it was not possible to extract them from the cartridge in order to analyse further. Visual analysis indicated that the blockage occurred at the site of mixing. It is believed that the desired iron oxide may have formed; however, due to the extremely short duration of reaction, this may not have formed Fe_3_O_4_ and could have been Fe_2_O_3_ or a mixture of both or, indeed, not in good quality if the mixing time was not long enough. Further work would be needed in order to develop a methodology to synthesise these cores, perhaps by diluting the reactants by an order of magnitude to stop the cartridge blocking upon mixing (therefore rendering it unusable). It was decided to proceed using iron oxide synthesised in the laboratory to determine whether the microfluidic setup could be used for the iterative coating steps to fulfil the question as to whether this was an appropriate technique for constructing the hybrids.

**Figure 2:**
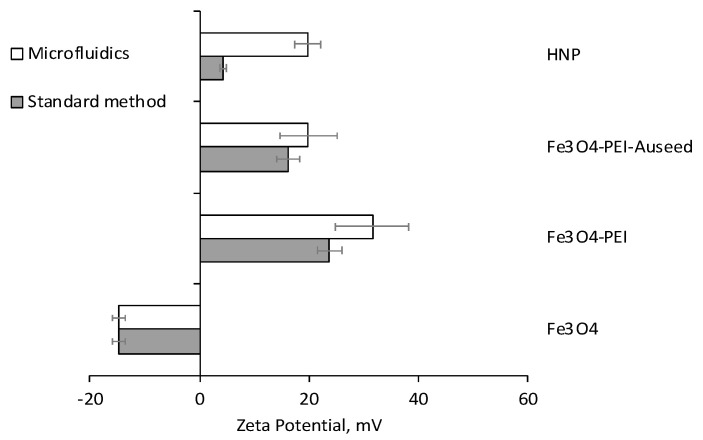
Zeta potential measurement of samples produced using standard laboratory synthesis and microfluidics (*n* = 3 ± SD).

The second step after iron oxide core synthesis in the fabrication of HNPs is surface coating of the core with PEI. In this step, it is expected that the negative surface charge of the iron oxide (due to sulphate association from its precursors) switches to a more positive value attributed to the cationic PEI if successful coating occurs. In those samples that had been mixed in the NanoAssemblr cartridge (Fe_3_O_4_ and PEI), it was observed that the surface charge changed from −14 to 32 mV (Figure 2), indicating that the coating had occurred. This finding was verified using Fourier transform infrared spectroscopy, where peaks were observed in both samples, which could only be attributed to the poly(ethylenimine) presence on the iron oxide surface. These included peaks at 2825 and 1500 cm^−1^ due to the presence of C-H groups, 1000 and 1500 cm^−1^ from the presence of N-H and from the presence of C-N (Figure S2). Interestingly the surface charge value was more positive than the 24 mV observed for the Fe_3_O_4_-PEI synthesised using the standard method, though the standard deviations overlapped, so this difference was not significant. To avoid any cartridge blockage observed in the iron oxide synthesis, this reaction was carried out under 100× dilution from the standard protocol; nevertheless, the coating did appear to be successful.

**Figure 3:**
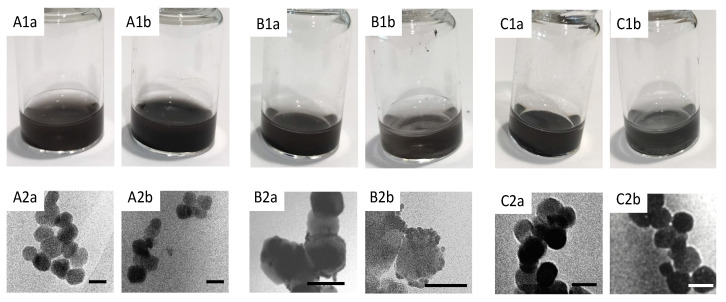
Images of (**A**) Fe_3_O_4_-PEI, (**B**) Fe_3_O_4_-PEI-Au seeds and (**C**) HNPs produced via a: standard methodologies and b: microfluidic manufacture as showing physical form in 1: photograph and 2: transmission electron micrograph (line denotes 50 nm scale).

Figure 3(A1a,A1b) shows the PEI-coated iron oxide obtained via both methodologies, which appear to be similar in appearance. Table 1 shows the hydrodynamic radius data collected for the iron oxide and after PEI coating. Here, it was observed that the uncoated iron oxide particles appeared to possess a large size of 2159 nm. However, due to the inherent magnetism of these particles, it is concluded that these data are measuring the size of clusters, and it is widely known that for magnetic particles, photon correlation spectroscopy is not the most appropriate method for size determination. With that in mind, the measurements obtained can only be used as indicative means to observe changes between coating steps. In agreement with the other literature for HNP synthesis [18], once PEI coating is achieved, the colloidal stability of the magnetic iron oxide increases, reducing aggregation, and hence, a notable drop in hydrodynamic radius is observed. Transmission electron microscopy was carried out to determine accurate particle size, as shown in Figure 3(A2a,A2b). Here, it is observed that the spherical particles possess a similar size of around 55 nm.

In the third synthetic step of HNP fabrication, gold seeds are attached onto the surface of the cationic Fe_3_O_4_-PEI particles. These seeds act as anchor points to encourage the complete coating in subsequent steps where chloroauric acid is reduced onto their surface. The 2 nm gold seeds possess a very slight electronegativity, which, after attachment, results in an overall reduction in the positivity of the precursor Fe_3_O_4_-PEI surface charge. Here, it was observed that after microfluidic mixing of the gold seed solution with the PEI-coated iron oxide to form Fe_3_O_4_-PEI-Au seeds, a reduction in zeta potential occurred from 32 down to 20 mV. This mapped well onto the expectations from the standard synthetic protocol, where a reduction from 24 mV of the Fe_3_O_4_-PEI down to 16 mV for the Fe_3_O_4_-PEI-Au seeds was observed. The hydrodynamic radius measurements carried out (Table 1) showed no change in particle size after attachment of the gold nanoseeds, which is to be expected as the presence of these seeds is unlikely to tip the balance in the colloidal stability towards further aggregation. In common with the Fe_3_O_4_-PEI-Au seeds synthesised using the standard procedure, the particle appearance in solution looked similar (Figure 3(B1a,B1b)).

However, the TEM images looked different, with a more ‘bobbly’ appearance of those particles manufactured using microfluidic mixing when compared with the standard synthesis (Figure 3(B2a,B2b)). When the Fe_3_O_4_-PEI and gold seeds are mixed in a round-bottom flask with stirring for 30 min and subsequently magnetically separated, it is often observed upon magnetic separation for the washing step that the supernatant solution possesses a red hue, indicating that not all the seeds have been attached. However, when the microfluidic manufactured particles were magnetically separated from the solution, this red hue was not observed, and the supernatant was colourless, thus indicating that more seed attachment occurred using the superior mixing of microfluidics, despite such mixing duration (72 s) being in seconds rather than hours. This phenomenon is reflected in the TEM image, where more gold seeds were evident (Figure 3(B2b)). This step was also carried out at 100× dilution compared to the standard protocol.

**Figure 4:**
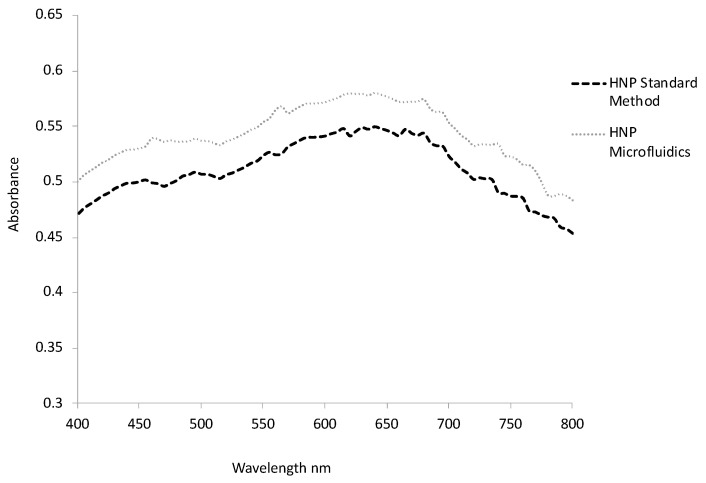
UV-vis spectroscopy of HNPs produced by standard methods and microfluidics. Samples scanned in deionised water over 400–800 nm.

The final step in hybrid formation is to reduce chloroauric acid onto the particle surface, which results in a complete coating. In this step, we expect a further reduction in the overall zeta potential if the complete coat is present due to the presence of a greater level of gold (and complete shielding of the cationic PEI layer) with its very slight electronegativity. Interestingly, those particles produced using the standard methods exhibited a greater reduction in zeta potential down to 4 mV compared with those produced from microfluidic mixing for which no change in zeta potential was observed. It is believed that the greater level of seeding on the Fe_3_O_4_-PEI-Au seeds produced from microfluidic mixing had almost coated the particles entirely with the further iterations of coating, perhaps leading to a thicker coat, but not resulting in an overall change in surface charge. UV–vis spectroscopy confirmed that both HNPs possessed a lambda max of around 650 nm, which is indicative of their surface plasmon resonance. In line with that of colloidal cold. The similarity of lambda max value, also indicated that both the particles were of a similar size range, as gold nanoparticles exhibit greater red-shifting as their size (and surface area) increases (Figure 4). The particles’ physical appearance looked identical (Figure 3(C1a,C2b)) in solution and the size under TEM looked consistent across both methods (Figure 3(C2a,C2b)), which was observed to be around 60 nm.

When the size was measured for these particles using photon correlation spectroscopy, large clusters were once again forming with sizes of 1050 nm for the standard procedure and 1347 nm after microfluidic manufacture (Table 1). As gold nanoparticles form at a larger scale, there is a great tendency to aggregate, and it is presumed this is the reason for the cluster formation. The HNPs were analysed for their metal content to fully determine whether their structural composition was similar. Here, it was observed that the Fe/Au ratio of those HNPs synthesised using the standard protocol was 1:0.6 and for those produced using the NanoAssemblr 1:0.7. The attachment of more gold seeds theoretically may have contributed to the increased gold content of the final HNPs.

This finding indicated that the particles formed using the microfluidic technique were structurally similar to those fabricated using the standard protocol. Thermogravimetric analysis of the HNPs in Figure S3 displays mass loss over three main temperature ranges over time, which are the main heating stage from 25 to 600 °C, the 600 °C hold and the cooldown from 600 to 200 °C. HNPs produced by both the standard method and microfluidic method show similar smooth mass loss profiles in the latter two. This uniform loss over time has been observed to occur at 273 °C in the literature, similar to our microfluidic-derived HNPs and, when coupled with the very similar heat flow profile, is evidence of their successful synthesis via microfluidics [23]. The temperature ramp from 25 to 600 °C, for both HNPs, is <1% and likely attributed to trace surface water. There is a small mass increase for those particles produced through the standard procedure. This is likely to be due to iron located at the surface or under a very thin/nonuniform layer of gold. The subsequent oxidation as the temperature rises would cause a mass increase up until a critical temperature prior to a “burn-off”. This is likely a batch quality event and was not observed in the microfluidic HNPs. Further highlighting the batch-to-batch quality variability of the standard procedures for HNP synthesis and the advantages in HNP quality that an automated microfluidics system would provide. These exciting findings have large implications for the ability to rapidly and continuously manufacture the HNPs, which will undoubtedly benefit their clinical translation.

**Figure 5:**
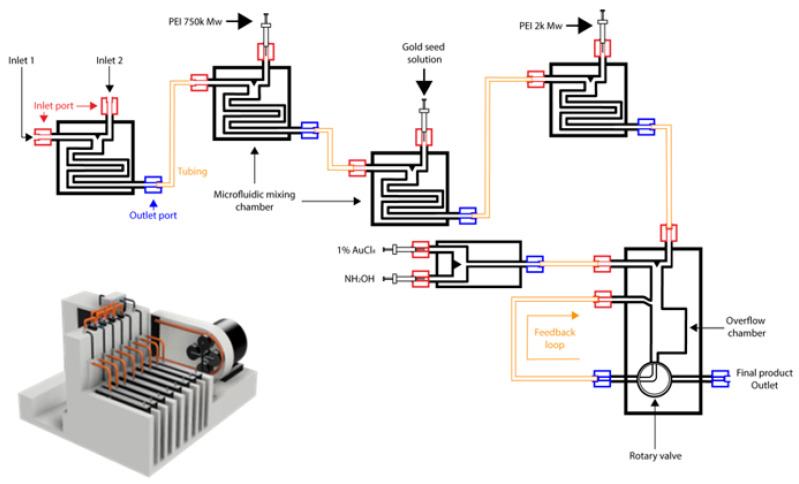
Proposed microfluidic setup for continuous manufacture of hybrid nanoparticles showing schematic representation and computer-aided design prototype.

Although syringe drivers were used to introduce the reactants together in this short proof-of-concept study, it is envisaged that the particles would be able to undergo large-scale continuous manufacture using a microfluidics system by altering the inlet ports to allow for fluid flow at defined flowrates into the mixing chambers, and configuring further inlet valves in a linear fashion such as those shown in Figure 5, in order to develop large-scale manufacture for HNPs.

The output of HNPs produced under standard lab procedures was approximately 2.5 times higher than with the microfluidics system (when factoring in the initial 100× dilutions). Obviously, further optimisation would be required to configure such a plant, with consideration of pressure, flow rates, mixing times and shear. However, given that the standard protocol for these particles can take up to 1 week to produce the particles and microfluidics can do this in minutes with the potential to move towards continuous manufacture, these findings are very exciting. In this study, the iron oxide nanoparticles and gold seeds were manufactured in the laboratory and not produced using microfluidics. It would be desirable if these could be produced within the microfluidic plant, allowing for full synthesis without blocking the cartridges, and more work is being carried out in our laboratory to investigate this. However, if that is not possible, both components can be made at a scalable volume, and this would not hinder the progression of the desired continuous manufactured system.

The final consideration to make is the concentration of particles produced. In all the microfluidic reactions (after the failed Fe_3_O_4_ attempts), the reactants were introduced at 100× lower concentration than in the standard protocol; however, the magnetic core of the HNPs allows them to be magnetically separated easily from solution and concentrated for use or stored in solid form. The water-saving advantage of the benchtop method is mitigated by the large volumes of water required during the frequent washes and magnetically aided separation steps. Therefore, it is not expected that this reduced concentration in the microfluidic production detracts from the benefits of the total 2.5-fold faster process of PEI coating, gold seeding and outer shell gold coating attributed to the superior mixing of the microfluidics system. Further optimisation of these protocols may also allow for an increase in reagent concentrations, further improving the production rate. In contrast to the benchtop method, where human input only occurs at key points, reducing the HNPs with the NanoAssemblr system at a 2.5-fold rate would require continuous manual handling of an operator. However, further optimisation for higher reagent concentrations and the implementation of syringe pump inputs and larger reservoir outputs would overall establish the microfluidics process as the superior method of synthesising HNPs from uncoated IONPs.

## 4. Conclusions

In this study, we report the potential of microfluidics as a mechanism for the scale up and manufacture of core-shell inorganic gold-coated iron oxide nanoparticles. This work highlights that the methodology requires optimisation, but in common with the liposome work in the field, it appears that microfluidics is an appropriate technique to use in the continuous manufacture of hybrid particles, bringing the possibility of their use in medical sciences a step further towards clinical translation.

## Figures and Tables

**Table 1:** Hydrodynamic diameter and metal content analysis of the hybrid nanoparticles.

Particle	Standard			Microfluidics		
	Metal Content Analysis mgmL^−1^		Size nm (±SD)	Metal Content Analysis mgmL^−1^		Size nm (±SD)
	Fe	Au		Fe	Au	
Fe_3_O_4_	-	-	2159 (154)	-	-	2159 (154)
Fe_3_O_4_-PEI	-	-	208 (7)	-	-	277 (189)
Fe_3_O_4_-PEI-Au_SEED_	-	-	201 (12)	-	-	796 (390)
HNP	15.71	9.85	1050 (350)	6.468	4.283	1347 (730)

## Data Availability

Data will be made available upon reasonable request.

## Supplementary Materials

The following are available online at www.mdpi.com/10.3390/nano11112976/s1, Figure S1: Precision NanoAssemblr^®^ Benchtop system with commercially available cartridges used, Figure S2: FTIR of Fe_3_O_4_-PEI nanoparticles prepared via (A) standard protocols and (B) microfluidics. Samples were run in freeze dried form with 64 scans, Figure S3: TGA analysis of HNPs prepared via (A) standard protocols and (B) microfluidics.

## References

[1] Pomerantseva E., Bonaccorso F., Feng X., Cui Y., Gogotsi Y. (2019). The future enabled by nanomaterials. Science.

[2] Guerra F.D., Attia M.F., Whitehead D.C., Alexis F. (2018). Nanotechnology for Environmental Remediation: Materials and Applications. Molecules.

[3] Anselmo A.C., Mitragotri S. (2019). Nanoparticles in the clinic: An update. Bioeng. Transl. Med..

[4] Muthu M.S., Wilson B. (2012). Challenges posed by the scale-up of nanomedicines. Nanomedicine.

[5] Longo J.P.F., Mussi S., Azevedo R.B., Muehlmann L.A. (2020). Issues affecting nanomedicines on the way from the bench to the market. J. Mater. Chem. B.

[6] Hua S., de Matos M.B.C., Metselaar J.M., Storm G. (2018). Current Trends and Challenges in the Clinical Translation of Nanoparticulate Nanomedicines: Pathways for Translational Development and Commercialization. Front. Pharmacol..

[7] Carugo D., Bottaro E., Owen J., Stride E., Nastruzzi C. (2016). Liposome production by microfluidics: Potential and limiting factors. Sci. Rep..

[8] Kotouček J., Hubatka F., Mašek J., Ku-lich P., Velínská K., Bezděková J., Fojtíková M., Bartheldyová E., Tomečková A., Stráská J. (2020). Preparation of nanoliposomes by microfluidic mixing in herring-bone channel and the role of membrane fluidity in liposomes formation. Sci. Rep..

[9] Lou G., Ganderluzzi G., Woods S., Roberts C.W., Perrie Y. (2019). A novel microfluidic-based approach to formulate size-tuneable large unilamellar cationic liposomes: Formulation, cellular uptake and biodistribution investigations. Eur. J. Pharm. Biopharm..

[10] Khadke S., Roces C.B., Donaghey R., Giacobbo V., Su Y., Perrie Y. (2020). Scalable solvent-free production of liposomes. J. Pharm. Pharmacol..

[11] Liu F., Yin K.L., Liu H. (2021). A microfluidic synthesis method for preparation and regulation of 3-aminophenol formaldehyde resin spheres. React. Funct. Polym..

[12] Niculescu A.-G., Chircov C., Bîrcă A.C., Grumezescu A.M. (2021). Nanomaterials Synthesis through Microfluidic Methods: An Updated Overview. Nanomaterials.

[13] Webb C., Forbes N., Roces C.B., Ander-luzzi G., Lou G., Abraham S., Ingalls L., Marshall K., Leaver T.J., Watts T.A. (2020). Using microfluidics for scalable manufacturing of nanomedicines from bench to GMP: A case study using protein-loaded liposomes. Int. J. Pharm..

[14] Brennan G., Bergamino S., Pescio M., Tofail S.A.M., Silien C. (2020). The Effects of a Varied Gold Shell Thickness on Iron Oxide Nanoparticle Cores in Magnetic Manipulation, T1 and T2 MRI Contrasting, and Magnetic Hyperthermia. Nanomaterials.

[15] Oluwasamni A., Manzur A., Aldebasi M.H.M., Elsini R.S., Albusai M.K.A., Haxton K., Curtis A., Hoskins C. (2017). Diels Alder-mediated release of gemcitabine from hybrid nanoparticles for enhanced pancreatic cancer therapy. J. Control Release.

[16] Malekigorji M., Alfahad M.A.M., Kong Thoo Lin P., Jones S., Curtis A., Hoskins C. (2017). Thermally triggered theranostics for pancreatic cancer therapy. Nanoscale.

[17] Ding X., Li D., Jiang J. (2020). Gold-based Inorganic Nanohybrids for Nanomedicine Applications. Theranostics.

[18] Barnett C., Gueorguieva M., Lees M., Darton R., McGarvey D., Hoskins C. (2012). Effect of hybrid composition on physicochemical properties and morphology of iron oxide-gold nanoparticles. J. Nanopart. Res..

[19] Zhang W., Tianzhao Z., Zhao X., Yuan Y., Miao F., Li W., Ji S., Huang X., Chen X., Jiang T. (2020). Microfluidic Synthesis of Multimode Au@CoFeB-Rg3 Nanomedicines and Their Cytotoxicity and Anti-Tumor Effects. Chem. Mater..

[20] Panariello L., Wu G., Besenhard M.O., Loizou K., Storozhuk L., Thanh N.T.K., Gavriilidis A. (2020). A Modular Millifluidic Platform for the Synthesis of Iron Oxide Nanoparticles with Control over Dissolved Gas and Flow Configuration. Materials.

[21] James M., Revia R.A., Stephen Z., Zhang M. (2020). Microfluidic Synthesis of Iron Oxide Nanoparticles. Nanomaterials.

[22] Ahrberg C.D., Wook Choi J., Geun Chung B. (2020). Automated droplet reactor for the synthesis of iron oxide/gold core-shell nanoparticles. Sci. Rep..

[23] Mohammad F., Arfin T. (2014). Thermodynamics And Electrochemical Characterization of Core-Shell Type Gold-Coated Superparamagnetic Iron Oxide Nanoparticles. Adv. Mater. Lett..

