# Supplementary material for: Investigation into the Use of Microfluidics in the Manufacture of Metallic Gold-Coated Iron Oxide Hybrid Nanoparticles
Source: Nanomaterials (Basel). 2021 Nov 5;11(11):2976. doi: 10.3390/nano11112976 (PMC8622423; doi:10.3390/nano11112976)
Supplement: Supplementary file 1 [file nanomaterials-11-02976-s001.zip › nanomaterials-1408393-supplementary.pdf]

nanomaterials

Supporting Information

Investigation into the Use of Microfluidics in the Manufacture of Metallic Gold-Coated Iron Oxide Hybrid Nanoparticles

Adeolu Oluwasanmi [1], Ernest Man [2], Anthony Curtis [3], Humphrey H. P. Yiu [4], Yvonne Perrie [5] and Clare Hoskins [6,*]

Citation: Oluwasanmi, A.; Man, E.; Curtis, A.; Yiu, H.H.P.; Perrie, Y.; Hoskins, C. Investigation into the Use of Microfluidics in the Manufacture of Metallic Gold-Coated Iron Oxide Hybrid Nanoparticles. *Nanomaterials* **2021**, *11*, 2976. https://doi.org/10.3390/nano11112976

Academic Editor: Ovidiu Ersen

Received: 20 September 2021
Accepted: 03 November 2021
Published: 5 November 2021

Publisher's Note: MDPI stays neutral with regard to jurisdictional claims in published maps and institutional affiliations.

Copyright: © 2021 by the authors. Licensee MDPI, Basel, Switzerland. This article is an open access article distributed under the terms and conditions of the Creative Commons Attribution (CC BY) license (http://creativecommons.org/licenses/by/4.0/).

[1] Department of Pure and Applied Chemistry, University of Strathclyde, Glasgow G1 1RD, UK; adeolu.oluwasanmi@strath.ac.uk (A.O.); ernest.man@strath.ac.uk (E.M.)
[2] School of Pharmacy and Bioengineering, Keele University, Keele ST5 5BG, UK; a.d.m.curtis@keele.ac.uk
[3] Chemical Engineering, School of Engineering and Physical Sciences, Heriot-Watt University, Edinburgh EH14 4AS, UK; h.h.yiu@hw.ac.uk
[4] Strathclyde Institute of Pharmacy and Biomedical Sciences, University of Strathclyde, Glasgow G4 0RE, UK; yvonne.perrie@strath.ac.uk
* Correspondence: clare.hoskins@strath.ac.uk; Tel.: +44-(141)-5482796

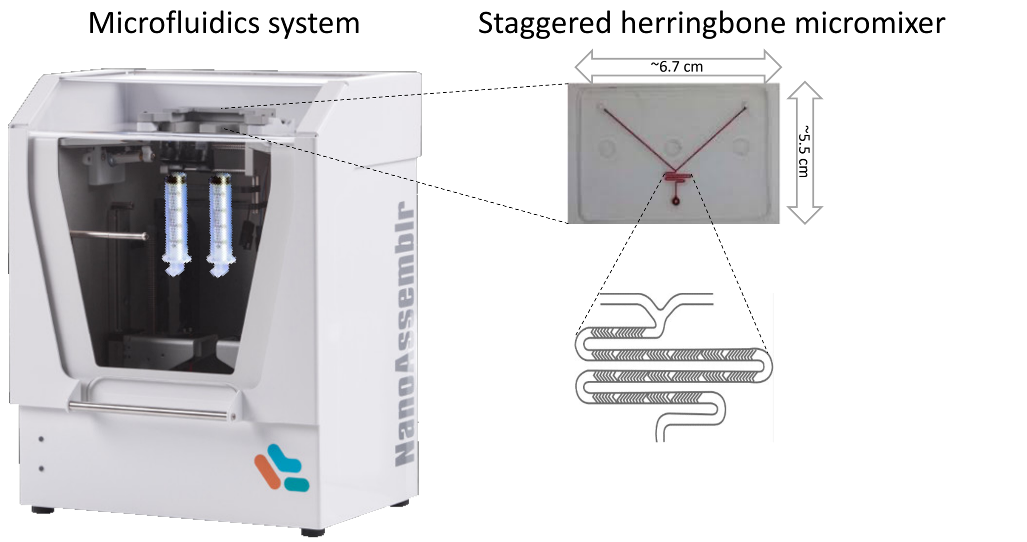

Figure S1: Precision NanoAssemblr® Benchtop system with commercially available cartridges used.

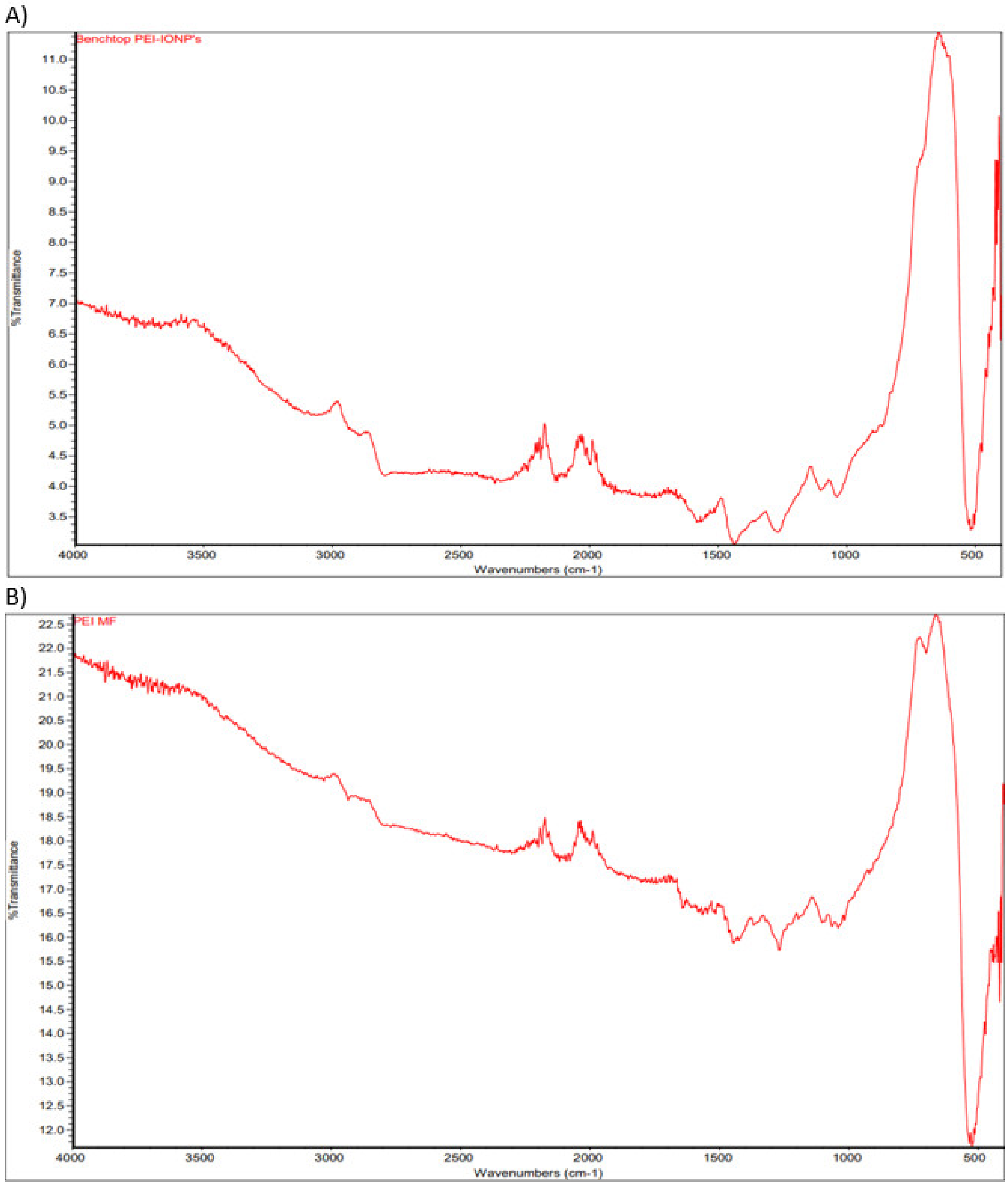

Figure S2: FTIR of Fe3O4-PEI nanoparticles prepared via (**A**) standard protocols and (**B**) microfluidics. Samples were run in freeze dried form with 64 scans.

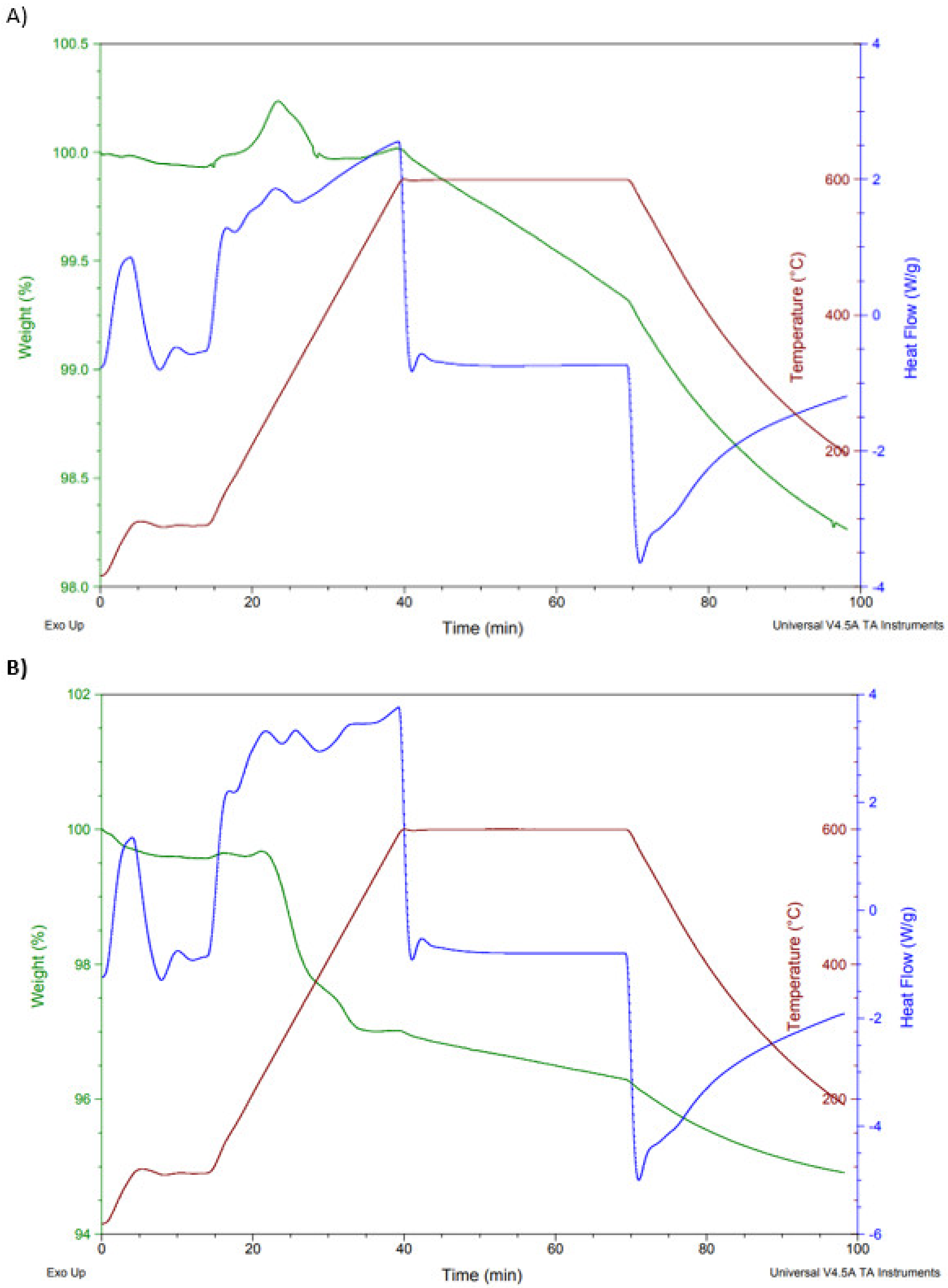

Figure S3. TGA analysis of HNPs prepared via (**A**) standard protocols and (**B**) microfluidics.
